# A New HEK293 Cell with CR2 Region of E1A Gene Deletion Prevents the Emergence of Replication-Competent Adenovirus

**DOI:** 10.3390/cancers15245713

**Published:** 2023-12-05

**Authors:** Xueqi Lian, Xiaoyan Zhao, Jingjing Zhong, Chenglin Zhang, Yongchao Chu, Yaohe Wang, Shuangshuang Lu, Zhimin Wang

**Affiliations:** 1 National Center for International Research in Cell and Gene Therapy, Sino-British Research Centre for Molecular Oncology, School of Basic Medical Sciences, Academy of Medical Sciences, Zhengzhou University, Zhengzhou 450052, China; lianxueqi2048@gs.zzu.edu.cn (X.L.); zhaoxy2023@zzu.edu.cn (X.Z.); jjingzhong2023@zzu.edu.cn (J.Z.); zhangchenglin1995@gs.zzu.edu.cn (C.Z.); cyc@zzu.edu.cn (Y.C.); yaohe.wang@qmul.ac.uk (Y.W.); 2 Centre for Biomarkers & Biotherapeutics, Barts Cancer Institute, Queen Mary University of London, London EC1M 6BQ, UK

**Keywords:** E1A, CR2, 293 cell line, Replication-Competent Adenovirus

## Abstract

**Simple Summary:**

During high titer recombinant oncolytic adenovirus production, Replication-Competent Adenovirus (RCA) contaminants can be generated through recombination between oncolytic virus and host cells due to DNA sequence similarity. The HEK293 cell is a widely used cell line with the adenoviral genome present to compensate for early region 1A (E1A) function for E1A-deleted adenovirus. Therefore, RCA may present through homologous recombination between the virus genome and cell genome for recombinant adenovirus production; these RCA contaminants raise various safety concerns in clinics. Our study aimed to eliminate the production of RCA in adenovirus production by modifying the producer cells. We confirmed that the conserved region (CR) 2 deletion of the E1A gene in the producer HEK293 cell line could effectively prevent RCA formation without affecting the cell proliferation and adenovirus production, which provides safer clinical applications of oncolytic adenovirus.

**Abstract:**

Purpose: To eliminate the contaminants of Replication-Competent Adenovirus (RCA) during high titer recombinant oncolytic adenovirus production. Methods: At first, we detected E1A copy numbers of different sources of 293 cells using Q-PCR, and we screened a subclone JH293-C21 of the JH293 cell line (purchased from ATCC) with lower early region 1A (E1A) copy numbers and higher adenovirus production ability. Then, we deleted the conserved region (CR)2 of the E1A gene in this subclone using the CRISPR-Cas9 system and obtained a stable cell clone JH293-C21-C14 with lower E1A expression, but the RCA formation had no significant reduction. Then, we further deleted the CR2 of JH293-C21-C14 cells with the CRISPR-Cas9 system and obtained a strain of cells named JH293-C21-C14-C28. Finally, we detected the capacity for cell proliferation, adenovirus production, and RCA formation in the production of recombinant adenovirus. Results: The JH293-C21-C14-C28 cells had a similar cell proliferation ability and human adenovirus production as JH293-C21 cells. Most importantly, RCA production in JH293-C21-C14-C28 cells was lower than in JH293-C21 cells. Conclusion: Human adenovirus producer cell clone JH293-C21-C14-C28 with CR2 deletion can effectively prevent the RCA production of replication-competent oncolytic adenovirus; this will provide significant advantages in utility and safety in gene therapy.

## 1. Introduction

Oncolytic viruses (OVs) have been developed into a novel and prospective type of therapeutic virus since the first clinical trial with OVs, and are commonly used to selectively infect tumor cells, replicate, and further cause tumor cell death. OVs, including adenoviruses, vaccinia viruses, herpes simplex viruses, Coxsackie viruses, etc., are being combined with gene therapy to treat tumors. For example, Gendicine, which is based on oncolytic adenovirus type 5, was the first gene therapy drug. Human Adenoviruses (HAdVs) were discovered in 1953 [1], and more than 120 species-specific adenoviral serotypes have been identified in humans, mammals, birds, fish, and reptiles up to now [2]. AdVs are 90–100 nm non-enveloped icosahedral viruses with a linear, double-stranded DNA genome of about 26–48 kb [3,4]. They are divided into seven species (A–G), and more than 100 types are classified in human AdVs, including serotypes 1–52 and genotypes 53–103 [5,6]. Unlike animal AdVs that appear to be associated with clinically important diseases in animals and birds [7], HAdVs do not cause severe diseases in immunocompetent humans [8]. In particular, HAdVs serotypes 2 and 5 of species C, whose infection often causes mild upper respiratory tract infections in childhood, have been extensively studied and used as gene therapy vectors [9,10]. 

In past decades, more and more gene therapies were developed to treat hereditary genetic diseases, primarily monogenic ones, cancer, cardiovascular diseases, and infectious diseases. While many viruses have been engineered to be used as delivery vectors in gene therapies, AdVs, adeno-associated virus (AAV), and lentivirus (LV) are among the most popular [11]. AdVs have several advantages as delivery vectors over AAV and LV. Compared to AAV, AdV has a broader range of infective cell types because the cell receptor of AdV is expressed widely in many different cell types. In comparison, different serotypes of AAV are quick to infect specific cell types and tissues. Otherwise, AdVs can deliver up to 30 kb of transgene, and AAV can only deliver up to 4.7 kb of transgene [12]. In contrast to LV infection, AdV’s infection does not depend on cell cycle status and rarely involves the integration of viral genes into the host genome [13,14]. 

Of note, as wild-type AdVs may cause severe infections in immunocompromised people [15,16,17], recombinant AdVs, including replication-defective AdVs and conditionally replicating adenovirus such as oncolytic viruses, are used as gene therapy vectors in regenerative medicine and cancer therapy. Oncolytic adenoviruses are commonly designed to induce tumor cell death with the modification of viral gene deletion or insertion of the E1 region because the early region 1A (E1A) gene is the first gene expressed upon viral infection and is crucial for all subsequent viral gene expressions [18]. For most oncolytic AdVs, the conserved region (CR) 2 of the E1A gene is deleted, and the promoter of E1A is replaced with a tumor cell-specific promoter [19,20,21,22]. The CR2 region of the E1A gene inhibits retinoblastoma (Rb) binding to the E2F family transcription factors in infected cells, thereby inducing quiescent cells to progress into the S-phase, which is essential for viral replication [23]. Therefore, the CR2-deleted virus can only replicate in tumor cells that lack Rb, and not in normal cells with Rb.

Replication-Competent Adenovirus (RCA) production within oncolytic adenoviruses production is a significant safety concern in OV’s clinical applications. During high-titer recombinant AdV production, RCA contaminants can be generated through recombination between the oncolytic virus and host cells due to the overlap of their DNA sequences. HEK293 cell is a widely used cell line for recombinant AdV production. The adenoviral genome 1–4344 presents in this cell line and compensates for E1A function for E1A-deleted AdVs. Therefore, RCA may present through homologous recombination between the virus and the cell genome [24]. Many investigators have minimized the homologous sequences between the viral vector and the transfected E1 DNA in packaging cells to avoid RCA production. One group of researchers developed a new helper cell line, PER, with no sequence overlap with a matched AdVs vector [25]. It eliminated RCA generation through homologous recombination compared with HEK293 cells. However, it can only be used for the propagation of matched AdVs. Another group of researchers described a HeLa-based cell line called GH329 that stably expresses a shorter E1 locus from a promoter derived from a phosphoglycerate kinase gene to eliminate the overlap sequence with the genome of AdVs [26]. This cell line also eliminated the production of RCA. However, using tumor cell line HeLa increases the safety concerns of host cell DNA residues of the final AdV production.

Here, we developed a new JH293-C21-C14-C28 cell line with the CR2 region of E1A gene deletion based on a clonal JH293-C21 cell of the JH293 cell line (purchased from ATCC). We found that JH293-C21-C14-C28 cells have similar cell proliferation ability and HAdV production ability to JH293-C21 cells, and most importantly, the RCA production in JH293-C21-C14-C28 cells was lower than that in JH293-C21 cells. This confirmed that the deletion of the CR2 in producer 293 cells could effectively eliminate the production of RCA in recombinant AdV production. 

## 2. Materials and Methods

### 2.1. Cell Culture and Reagents

JH293 and Hela cells were purchased from the American Type Culture Collection (ATCC, Mansas, VA, USA. Catalog number: CRL-1573) and were cultured in Dulbecco’s modified Eagle medium (DMEM, GIBCO, ThermoFish Scientific, Waltham, MA, USA) supplemented with 10% Fetal bovine serum (Biological Industries, Beit Haemek, Israel). HEK293 cells were purchased from the cell bank of the Chinese Academy of Sciences (CAS, Shanghai, China. Catalog number: GNHu43), and were grown as above. All cells were cultured in a humidified incubator with a 5% CO_2_ atmosphere at 37 °C. These cells were characterized by short tandem repeat (STR) markers and were further confirmed to be mycoplasma-free.

### 2.2. Cell Proliferation Assay

Cells were seeded into 24-well plates at 10,000 cells/well in triplicate the day before and were counted on the second to the seventh days as follows: the medium was discarded and the cells were rinsed with PBS (GIBCO, ThermoFish Scientific, Waltham, MA, USA), then 100 μL TrypLE Select Enzyme solution (GIBCO, ThermoFish Scientific, Waltham, MA, USA) was added to the well for 1 min and 900 μL complete medium was added to terminate the reaction. The cells were diluted two times with Trypan blue Solution (Sigma Co., St. Louis, MO, USA) and counted with Counter Star [Ruiyu, Shanghai, China].

### 2.3. Screening of Monoclonal Cell

Cells were seeded into 96-well plates at a density of less than 1 cell per well and the single visible clone was marked after culturing for 24–48 h. Cells were further cultured until they could be transferred to 24-well plates and further expansion could be conserved and identified.

### 2.4. CRISPR/Cas9 Mediated Deletions and Sequencing

Six single-guide RNA (sgRNA) targets for the E1A exon were designed using the MIT CRISPR Design website (http://crispr.dfci.harvard.edu) (accessed on 23 July 2020), and the primers were listed in the Supplementary Data (Table S1). Only high-score guide RNAs were used to minimize the potential off-target effects of the guide RNA. Guide RNAs were obtained from Shangya (Shanghai, China). Then, sgRNAs were cloned separately into PX459 plasmids containing Cas9. Cells were seeded into 6-well plates the day before and transfected with PX459 plasmids containing sgRNAs by using polyethyleneimine (PEI, Polysciences, Warrington, PA, USA) according to the manufacturer’s instructions. The 0.5 μg/mL puromycin (Solarbio, Beijing, China) was added to the cells after transfecting for 48 h. The cell screening was followed by monoclonal selection and further expansion.

PCR primers of the E1A gene containing the sgRNA target sequence were synthesized, and then DNA extracted from the screened monoclonal cells was used as the template for the PCR reaction, and the products were analyzed using Shangya (Zhengzhou, China).

### 2.5. Western-Blot Assay

Cells were harvested and rinsed with PBS and were lysed in RIPA lysis buffer (Beyotime, Shanghai, China) containing 1 mM Phenylmethanesulfonyl fluoride (PMSF) and centrifuged according to the manufacturer’s description. The supernatant concentration was detected by using the BCA Protein Assay Kit (Solarbio, Beijing, China), and then the supernatant was boiled for 5 min after adding the protein loading buffer (cwbiotech, Beijing, China). Protein samples were separated with sodium dodecyl sulfate polyacrylamide gel electrophoresis (SDS-PAGE) and transferred to polyvinylidene fluoride membranes (PVDF, Millipore, Darmstadt, Germany). The membrane was incubated with a primary antibody overnight at 4 °C after blocking with 5% non-fat milk for 1 h at room temperature, and then the membrane was incubated with a secondary antibody for 1 h at room temperature. The band signals were visualized and quantified using the Amersham Imager 600 (GE, Pittsburgh, PA, USA). The following antibodies were used in this study: anti-E1A (Abcam, Cambridge, MA, USA), anti-GAPDH (ProteinTech Group, Chicago, IL, USA).

For all Western blot figures, the intensity ratio of each band was processed with Image J software. This article’s bar charts or line charts were made with GraphPad/Prism 6.

### 2.6. E1A Gene Copies Number Tests

The copy number of the E1A gene in 293 cells was evaluated using qPCR. qPCR primers were designed by primer premier 5 software, and the sequences were listed in Supplementary Data (Table S2). First, the absolute values of copy numbers of E1A and albumin genes were quantified based on the reference material standard curve. The quantitative reference materials we used here were PCR products containing known copy numbers of the amplified fragments. The preparation process was as follows: perform PCR using the JH293 cell (purchased from ATCC) genome as a template to obtain E1A and Albumin amplified fragments, respectively, use gel recovery, and then use Nanodrop to measure the content. PCR primers of two genes were synthesized to obtain a template of the standard curve: albumin gene (two alleles, gene-specific reference): Albumin-PCR forward 5′-TTGCTGTCATCTCTTGTGG-3′ and reverse 5′-ATTATTCAGATTTTGGAAGTGC-3′; E1A gene: E1A-PCR forward 5′-GGCGTAACCGAGTAAGATTTG-3′ and reverse 5′-TAGCCCACGGCGCATTA-3′. Then, the absolute copy number was calculated based on the length of the PCR products. A different dilution was then used to establish a qPCR standard curve to calculate the absolute copy number of the E1A and albumin in cell-extracted DNA, as described previously [27].

### 2.7. HAdVs and Replication

The HAdV-Cre non-replicative virus was purchased from Vector Biolabs and propagated in our laboratory. Wild-type human adenovirus serotype 5 (HAdV-5) was described previously [28], and HAdV-TD was designed as in the previous study [29]. Human adenovirus serotype 11 (HAdV-11) was a kind gift from Daniel Stone and André Lieber (University of Washington, Seattle, WA, USA). For replication, HEK293 cells were infected with these viruses and then were harvested after 72 h and purified with a CsCl step gradient ultracentrifugation.

### 2.8. HAdVs Titration

Serial 10-fold dilutions of the HAdVs supernatant were made, and 10 μL of each dilution was inoculated into 12 parallel wells of 96-well plates, which were seeded 2000 cells/well the day before. The plates were incubated for 10 days at 37 °C in a 5% CO_2_ atmosphere. The plates were checked under a light microscope for cytopathology, and the TCID50 (50% tissue culture infective dose) was calculated using the Reed–Muench mathematical method [30].

### 2.9. DNA Extraction

The DNA of cells was extracted by using a mini column-based DNA isolation kit (BIOMIGA, San Diego, CA, USA) according to the manufacturer’s protocol. The DNA of the virus was extracted by using phenol-chloroform and 3 M pH5.2 NaAc (Beyotime, Shanghai, China) according to the manufacturer’s manual. Then, DNA concentration was determined spectrophotometrically at OD 260 nm.

### 2.10. Real-Time PCR

SYBR real-time PCR. The assay was performed using ChanQ Universal SYBR qPCR Master Mix (Vazyme, Nanjing, China); the optimal PCR conditions to amplify the Albumin and E1A gene were carried out as follows: initial denaturation and polymerase activation were performed at 95 °C for 30 s, the signal was detected during another 40 cycles (95 °C/10 s, 60 °C/30 s), and the melting curve analysis was 95 °C/15 s, 60 °C/60 s, and 95 °C/15 s.

Taqman real-time PCR. The StepOne Plus Real-Time PCR system (Applied Biosystems, Carlsbad, CA, USA) was used for the assay. The DNA extracted from the RCA standard or HAdVs-infected cells was dissolved in 20 μL of distilled H_2_O. Then, 10 μL of the DNA sample was used as a template in a subsequent real-time quantitative PCR with 0.5 μM of each primer, 0.16 μM TaqMan probe, and 25 μL of TaqMan universal PCR master mix (Applied Biosystems, Carlsbad, CA, USA). The cycling conditions were 50 °C for 2 min, 95 °C for 2 min, 95 °C/3 s, and 60 °C/30 s for a total of 40 cycles. The sequences of the primers were as follows: Ad5dE1-forward, 5′ TCCGGTCCTTCTAACACACCTC 3′ and Ad5dE1-reverse, 5′ ACGGCAACTGGTTTAATGGG 3′. The probe contains a fluorescent reporter dye, FAM, attached to the 5′ end of the oligonucleotide, and a nonfluorescent quencher, TAMRA, at the 3′ end. Ad5dE1-probe: 5′ FAM-TGAGATACACCCGGTGGTCCCGC-TAMRA 3′.

### 2.11. RCA Test

Before RCA testing, different cells were seeded in 6-well plates at 5×10^5^ cells/well; the medium was replaced with 2 mL fresh medium containing HAdV-cre non-replicable virus with or without the replicable HAdV-5 seed virus at MOI = 3 after culturing for 1 day. The plates were incubated for 3 days, and then the medium and cells were harvested. The harvested samples were prepared with three cycles of freezing and thawing and then centrifuged for 5 min. The supernatant was detected for particle concentration. The virus was diluted with the excipient solution (20 mM Tris, 25 mM NaCl, 2.5% glycerol (*w*/*v*), PH 8.0) and incubated for 15 min at room temperature; the absorbance value at 260 nm was measured. 

We carried out this experiment according to previous reports [31] (PCT/US2007/026206) and made some changes. Briefly, Hela cells were seeded in 24-well plates at 1 × 10^5^ cells/well, and the equal virus particles were mixed with medium containing 2% FBS and then added to the plate. At day 1 and day 3 after infection, the medium and cells were harvested and the supernatant was obtained through three cycles of freezing and thawing. One-fourth of the cleared lysates from day 3 were used to infect Hela cells seeded the day before as the second infection. On day 1 and day 3 after the second infection, the cell lysates were harvested as the preceding step. The third infection was carried out according to the previous infection.

### 2.12. Statistical Analysis

Data were presented as the mean ± standard deviation. The difference was statistically significant at a *p*-value < 0.05, determined using the *t*-tests.

## 3. Results

### 3.1. Different Sources of 293 Cell Have Different Copy Numbers of E1A

**Figure 1:**
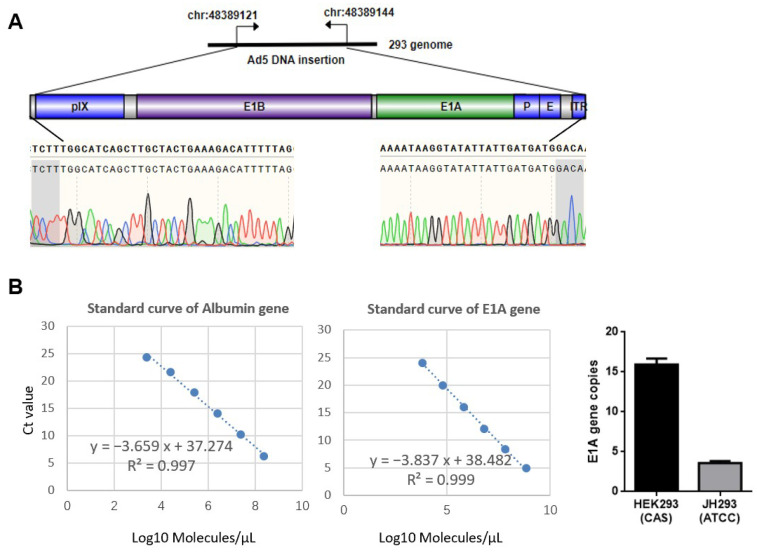
Early region 1A (E1A) copy number of different sources of 293 cell lines: (**A**) The illustration of the HAdV-5 DNA insertion site in 293 cell genomes. Below is shown the partial sequence of HAdV-5 DNA inserted into 293 cell genomes. Gray represents the partial sequence of the 293 cell genome. Green single peak represented Adenine(A), blue single peak represented Cytosine (C), black single peak represented Guanine(G)and red single peak represented Thymine (T). (**B**) E1A gene copy numbers were detected using Taqman qPCR. Molecules of Albumin and E1A were calculated according to the respective standard curve (scatter plot), with E1A copy number per cell = E1A molecules/(albumin molecules) × 2. The 2-fold factor reflects the presence of two alleles of the albumin gene. The number of molecules = (χ ng × 6.0221 × 10^23^ molecules/mole)/[(N × 660 g/mole) × 1 × 10^9^ ng/g], where χ is the amount of dsDNA (ng), N is the length of dsDNA, and 660 g/mole is the average mass of 1 bp dsDNA.

In order to delete the CR2 region of E1A in the 293 cell line thoroughly, we detected the E1A copy number of different sources of 293 cells using qPCR. First, we identified the location of this gene in 293 cells and compared it with the wild-type HAdV-5 genome (Figure 1A). Then, the E1A copy numbers were assessed using qPCR. We found that the cell line JH293 (purchased from ATCC) had a lower copy number of E1A than that bought from the cell bank of the Chinese Academy of Sciences (Figure 1B), and the E1A copy number of the cell line from ATCC was similar to the previous report [32]. Therefore, we chose the JH293 cell line to delete the CR2 region of E1A.

### 3.2. Selection of JH293 Single Clone JH293-C21 with High AdV Production Ability

**Figure 2:**
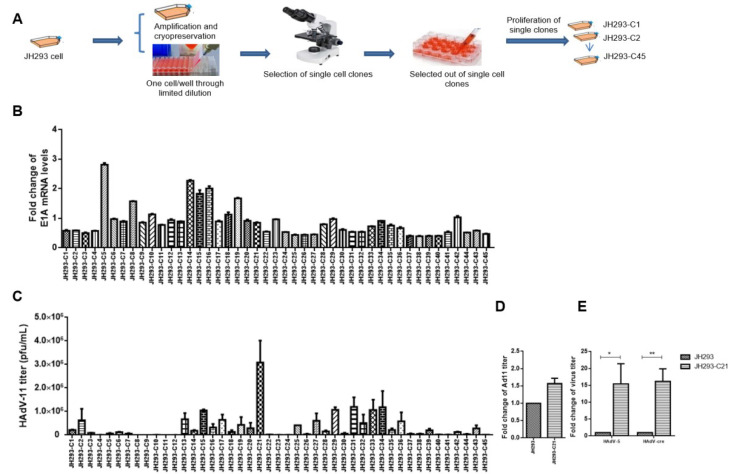
Selection of JH293 (purchased from ATCC) single clone with high HAdV production ability: (**A**) Screening of monoclonal cell selection. Cells were seeded into 96-well plates (<1 cell/well) by limited dilution, and then a single cell was observed and labeled under a microscope for further expansion. (**B**) RT-PCR detected the E1A mRNA expression level of monoclonal cells. GAPDH normalized the fold change of the mRNA level. (**C**,**D**) Virus titer was detected with a TCID50 assay. Monoclonal cells were infected with the Ad11 virus (MOI = 3), then cells and supernatants were collected together at 96 h post-infection, and the supernatant was conserved to use after three repeated freeze–thaws. (**D**) JH293 also normalized the fold change of the Ad11 titer. (**E**) JH293 also normalized the fold change of the HAdV-5 and HAdV-cre virus titer. The *p* values were obtained with one-tailed matched paired Student’s *t*-tests (* represents *p* < 0.05, ** represents *p* < 0.005).

Previous research has reported many genomic diversities in the 293 cell line; one of the reasons is that this cell line has been cultivated for decades in different laboratories [32,33]. In order to obtain a stable cell line with high AdV production ability, we selected single clones of JH293 cells (purchased from ATCC) using a limiting dilution assay (Figure 2A). Finally, we obtained 45 single clones and detected the E1A mRNA levels (Figure 2B) and HAdV-11 production ability (Figure 2C). We chose clone 21 (JH293-C21) cells for the E1A gene modification among all these clones because they can propagate Ad11 with high titers stably (Figure 2C,D). We also detected the production ability of HAdV-5 and HAdV-cre in JH293-C21. It still had a higher titer than JH293 (Figure 2E).

### 3.3. Construction of the CR2 Region of the E1A Knock-Out Cell Clone JH293-C21-C14-C28

**Figure 3:**
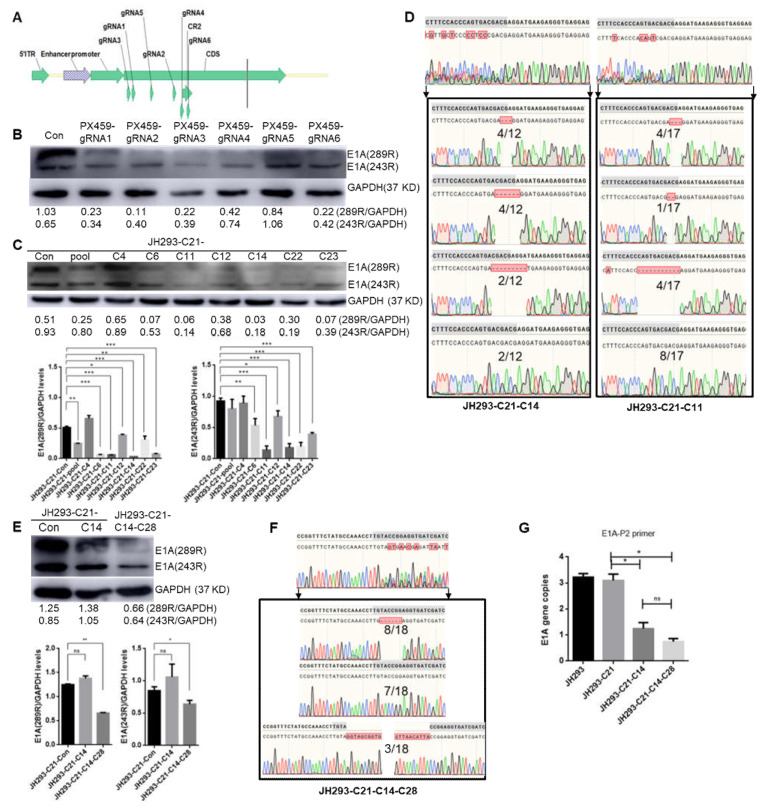
Construction of conserved region 2 (CR2) of the E1A gene knock-out JH293 cell clone: (**A**) gRNA position at E1A gene structure. (**B**) E1A gene expression level in JH293-C21 cells, which were transfected with plasmids PX459-sgRNA1, PX459-sgRNA2, PX459-sgRNA3, PX459-sgRNA4, PX459-sgRNA5, and PX459-sgRNA6, and cell lysates were harvested at 48 h after transfection and were detected with Western-blot. (**C**) E1A gene expression level was detected in monoclonal cells collected from JH293-C21 cells at 48 h after transfection with plasmid PX459-gRNA6 and sustained with puromycin (0.5 μg/mL). (**D**) Sequence of monoclonal JH293-C21-C14 cells and C11 cells. The DNA fragment containing the gRNA6 target of monoclonal cells was ligated to the pED-T vector and identified via sequence (the left showed sequences derived from the JH293-C21-C14 cell, and the right showed sequences derived from C11 cells). Bases with gray background indicate the gRNA6 targeted sequence. Green single peak represented Adenine (A), blue single peak represented Cytosine (C), black single peak represented Guanine (G) and red single peak represented Thymine (T). (**E**) Protein expression level in monoclonal JH293-C21-C14 cells and JH293-C21-C14-C28 cells. (**F**) Sequence of monoclonal JH293-C21-C14-C28 cells. The DNA fragment containing the gRNA4 target of monoclonal cells was ligated to the pED-T vector and identified via sequence. Bases with gray background indicate the gRNA4 targeted sequence. Green single peak represented Adenine (A), blue single peak represented Cytosine (C), black single peak represented Guanine (G)and red single peak represented Thymine (T). (**G**) E1A gene copy number detected with qPCR. The bars represent the SE. The *p* values were obtained with one-tailed matched paired Student’s *t*-tests (* represents *p* < 0.05, ** represents *p* < 0.005, *** represents *p* < 0.001, and ns represents no significant difference compared with the control group).

In order to delete the E1A gene, we designed several sgRNAs that target the CR2 region of E1A (Figure 3A). After the transfection of JH293-C21 cells with PX459-sgRNA separately, we checked the protein level of E1A and found that sgRNA6 had the highest knockout efficiency (Figure 3B). Then, we selected single clones in JH293-C21 cells that PX459-sgRNA6 transfected; we found the E1A protein level to be the lowest in clone 11 (C11) using Western blot, followed by clone 14 (JH293-C21-C14) (Figure 3C). Because there was more than one copy of the E1A gene in C11 and JH293-C21-C14 cells and we wanted to detect the situation of different copies of this gene, we amplified the gene region that was targeted by sgRNA6 through PCR and constructed it into plasmids to transfect competent cells, and obtained single plasmid clones. Then, we sequenced twelve clones derived from JH293-C21-C14 and seventeen from C11; we found that four clones had three nucleotides deleted, four clones had six nucleotides deleted, two clones had nine nucleotides deleted, and two clones were normal in JH293-C21-C14 cells (Figure 3D, Figure S1 and Table S3). At the same time, we found that four clones had three nucleotides deleted, one clone had two nucleotides deleted, four clones had twelve nucleotides deleted, and eight clones were normal in C11 cells (Figure 3D). These results suggested that the E1A gene was deleted more thoroughly in JH293-C21-C14 cells than in C11 cells. In order to delete the E1A gene further, we transfected Cas-sgRNA4 to JH293-C21-C14 cells and selected single clones; Clone 28 (JH293-C21-C14-C28) was the best clone with the lowest E1A gene expression (Figure 3E). Then, we detected the different copies of the E1A gene in JH293-C21-C14-C28 using the method described in JH293-C21-C14. We found that eight of eighteen clones had six nucleotides deleted, three of eighteen clones had fragment insertion, and seven of eighteen clones were normal (Figure 3F). Combined with 6 sgRNA target sites, we found all 18 clones were incomplete E1A genes (Table 1, Figure S2). We speculated that the presence of E1A protein in this cell line was CR2-region deficient. We also detected the E1A copy number in JH293-C21-C14-C28 using different primers that target the CR2 region or in front of it. We found that the E1A copy number in JH293-C21-C14 cells and JH293-C21-C14-C28 cells was lower than that in JH293-C21 cells (Figure 3G), and the E1A copy number detected by primers that targeted CR2 was lower than that detected by primers which do not target CR2 in CR2 deletion cells (Figure S3).

### 3.4. The JH293-C21-C14-C28 Clone Had Similar Cell Proliferation and AdV Production Ability to JH293-C21 Cells

**Figure 4:**
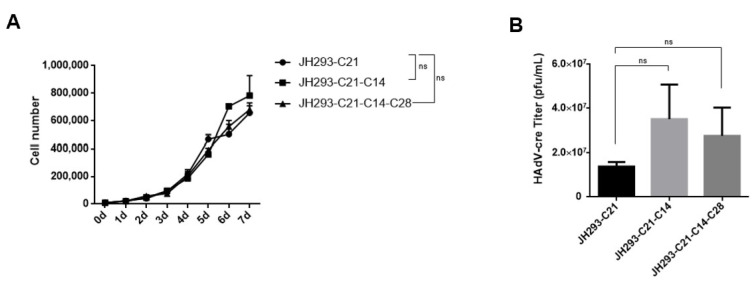
The proliferation ability and RCA production: (**A**) The proliferation ability of JH293-C21, JH293-C21-C14, and JH293-C21-C14-C28 cells. Cells were plated into 24-well plates, and cell number was calculated using counter star daily until day 7. (**B**) The virus replication capability of JH293-C21-C14-C28 cells. Cells were infected with replication-incompetent Ad-cre virus (MOI = 3) and were harvested at 96 h after infection. After three repeated freeze–thaws, the supernatants were used to carry out the TCID50 assay. The bars represent the SE. One-tailed matched paired Student’s *t*-tests obtained the *p* values. ns represents no significant difference compared with the JH293-C21 group.

After obtaining JH293-C21-C14-C28 cells, we detected their proliferation ability compared with JH293-C21 and JH293-C21-C14 cells and found a similar proliferation ability (Figure 4A). Then, we detected the propagation ability of the replication-incompetent HAdV-cre virus with E1 and E3 gene deletion in these cell lines and found that JH293-C21-C14-C28 cells have similar virus production ability to JH293-C21 and JH293-C21-C14 cells (Figure 4B).

### 3.5. The JH293-C21-C14-C28 Clone Prevents the Production of RCA in Recombinant AdVs

**Figure 5:**
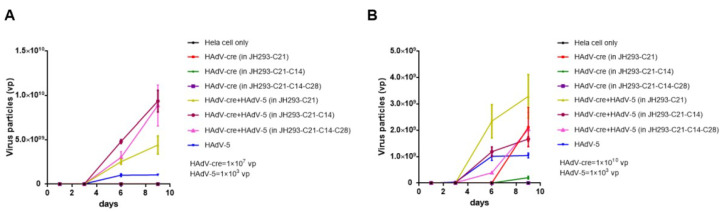
The production of RCA in recombinant HAdVs. Hela cells were infected with the 1 × 10^7^ (**A**) or 1 × 10^10^ vp (**B**) of HAdV-cre virus with or without wild-type HAdV-5 virus (HAdV-5 virus alone as the positive control). In Figure A, the Hela cell only, HAdV-cre (in JH293-C21), HAdV-cre (in JH293-C21-C14), and HAdV-cre (in JH293-C21-C14-C28) curves overlap with the X-axis, so they are not shown in the figure. In Figure B, the Hela cell only and HAdV-cre (in JH293-C21-C14-C28) curves overlap with the X-axis, so they are not shown in the figure. HAdV-cre (in JH293-C21), HAdV-cre (in JH293-C21-C14), and HAdV-cre (in JH293-C21-C14-C28) viruses were produced from JH293-C21, JH293-C21-C14, and JH293-C21-C14-C28 cell lines infected with HAdV-cre seed virus (MOI = 3) separately. Cells and supernatants were collected on day 1 and day 3 post-infection, and infection was repeated thrice. The DNA of the supernatant after three repeated freeze–thaws was extracted and used to perform the Taqman qPCR to detect the HAdV-cre virus particles.

Then, we detected the RCA production in Hela cells after we obtained the replication-incompetent HAdV-cre virus produced from JH293-C21, JH293-C21-C14, and JH293-C21-C14-C28 cells according to the previously reported method [31]. We found no RCA production in 10^7^ viral particles (vp) of the HAdV-cre virus from all three cell lines (Figure 5A). According to the safety standard, the RCA needed to be less than one in the 3 × 10^10^ vp of HAdVs for human gene therapy, so we also detected RCA production in 10^10^ vp of the HAdV-cre virus. We found RCA production in JH293-C21 and JH293-C21-C14 cells but not in JH293-C21-C14-C28 (Figure 5B). These results indicate that JH293-C21-C14-C28 cells could eliminate the production of RCA.

### 3.6. No E1A Gene Recombination Occurred in the Production of Oncolytic Adenovirus in the JH293-C21-C14-C28 Cell Line

**Figure 6:**
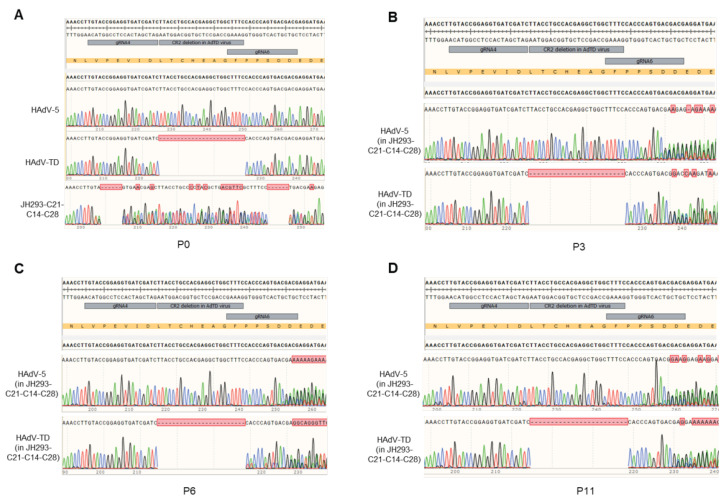
The sequence recombination in the CR2 of AdVs. (**A**) Sequence alignment of viruses and cells used. (**B**) Sequence alignment of 3 generations of virus produced in JH293-C21 cells (HAdV-5 in JH293-C21 and HAdV-TD in JH293-C21) and JH293-C21-C14-C28 cells (HAdV-5 in JH293-C21-C14-C28 and HAdV-TD in JH293-C21-C14-C28). (**C**) Sequence alignment of 6 generations of virus produced in JH293-C21 cells and JH293-C21-C14-C28 cells. (**D**) Sequence alignment of 11 generations of virus produced in JH293-C21 cells and JH293-C21-C14-C28 cells. Cells were infected with HAdV-5 or HAdV-TD virus (MOI = 3), and then cells and supernatants were collected on day 3 post infection; the titer was detected and then repeated as above. DNA was extracted and used to carry out Taqman qPCR detection. Green single peak represented Adenine (A), blue single peak represented Cytosine (C), black single peak represented Guanine (G)and red single peak represented Thymine (T).

As recombination may occur between the oncolytic virus and the host cell’s genome in the area with a similar sequence, we detected the occurrence of homologous recombination in the production of HAdVs in the JH293-C21-C14-C28 cell line. Our research group previously designed a tumor-selective oncolytic adenovirus (Ad-TD) with E1ACR2 and E1B19K deletion [29]. We used this virus and an HAdV-5 to carry out this part’s experiments (Figure 6A). We found that there was no sequence recombination in either HAdV-TD or HAdV-5 virus after three generations (Figure 6B), six generations (Figure 6C), and even eleven generations (Figure 6D) in JH293-C21-C14-C28 cells. These results further indicated that the JH293-C21-C14-C28 cell clone could reduce the homologous recombination in the CR2 region.

Furthermore, even if recombination occurred, the HAdVs still could not obtain a functional CR2 sequence in the JH293-C21-C14-C28 cells as this sequence was disrupted in the JH293-C21-C14-C28 cell genome. Therefore, the JH293-C21-C14-C28 cell line enhances the safety of oncolytic adenovirus production with CR2 deletion.

## 4. Discussion

Although recombinant AdVs have many disadvantages, such as high immunogenicity, short-term effects and complicated genome modification [34], they are still prevalent in killing cancer cells as oncolytic viruses and as COVID-19 or influenza vaccines in clinical contexts [35,36,37,38]. For safety concerns, it is crucial to eliminate RCA in high-titer recombinant AdV production for clinical use. Many efforts have been devoted to this field, and several different helper cell lines have been developed to resolve the RCA problem. However, as we mentioned in the Introduction, each of these helper cells has limitations. Therefore, we developed this new HEK293 cell line, JH293-C21-C14-C28, with the CR2 region of E1A gene deletion, to prevent RCA production. In our JH293-C21-C14-C28 cell line, with CR2 deletion, the homologous recombination rate between the cell genome and the virus genome will decrease dramatically. Even if the homologous recombination occurred between the cell genome and the virus genome in the case that the homologous 5′ and 3′ arm were still there, the homologous recombination CR2 from the cell genome to the virus genome is incomplete and cannot help the virus to replicate in normal cells with Rb expression.

Our new helper cell line, JH293-C21-C14-C28, has lower E1A protein expression than its parental cell line, JH293-C21 (Figure 3E), and we found no complete E1A copy in this cell line through plasmid clone construction and first-generation sequencing (Table 1). Although the level of E1A protein was low and the CR2 region of E1A is lost, the cell proliferation and AdV production ability of JH293-C21-C14-C28 cells were not eliminated and the RCA production was reduced. Most importantly, as the CR2 region of E1A is thoroughly lost in JH293-C21-C14-C28 cells, the produced RCA will be the CR2 region deleted and can only propagate in Rb-deficient tumor cells even if homologous recombination occurs between the AdVs and the JH293-C21-C14-C28 cell genome. Therefore, the JH293-C21-C14-C28 cell is a beautiful tool cell for the production of recombinant HAdVs.

## 5. Conclusions

This study showed that the deletion of CR2 in JH293-C21-C14-C28 cells could prevent RCA formation without affecting the proliferation rate and the HAdVs’ yield of JH293-C21-C14-C28 cells. These data supported using JH293-C21-C14-C28 cells as producer cells for HAdV production in clinical applications.

## Figures and Tables

**Table 1:** Change of E1A gene in JH293-C21-C14-C28 cells.

Mutation Type				
2#1	Clones of T vector	Change of gRNA6 situation	Change of gRNA4 situation besides gRNA6 situation	percentage
2#2	T1/T5/T8/T9/T11/T14/T20	6 bases deletion	No change	7/18
2#3	T2/T3/T4/T7/T10/T12/T15/T17	9 bases deletion	6 bases deletion	8/18
Total of E1A mutation: 18/18	T6/T13/T18	9 bases deletion	194 bases insertion	3/18

## Data Availability

All data presented are available from the authors on request.

## Supplementary Materials

The following supporting information can be downloaded at: https://www.mdpi.com/article/10.3390/cancers15245713/s1, Table S1: Sequences of gRNA targeting E1A gene; Table S2: Primers for E1A copy numbers determination; Table S3: Change in E1A gene in JH293-C21-C14 cells; Figure S1: The sequence of E1A genes in JH293-C21-C14 cells; Figure S2: The sequence of E1A genes in JH293-C21-C14-C28 cells; Figure S3: Detection of E1A copy numbers using different primers.
