# Supplementary material for: A New HEK293 Cell with CR2 Region of E1A Gene Deletion Prevents the Emergence of Replication-Competent Adenovirus
Source: Cancers (Basel). 2023 Dec 5;15(24):5713. doi: 10.3390/cancers15245713 (PMC10742158; doi:10.3390/cancers15245713)
Supplement: Supplementary file 1 [file cancers-15-05713-s001.zip › cancers-2696071-supplementary.pdf]

cancers MDPI

Supplementary

A New HEK293 Cell with CR2 Region of E1A Gene Deletion Prevents the Emergence of Replication-Competent Adenovirus

Table S1. Sequences of gRNA targeting E1A gene.

Name	Sequence
gRNA1	CCAGTCTTTTGGACCAGCTG
gRNA2	AGCAGCCGGAGCAGAGAGCC
gRNA3	CGGAGGTGTTATTACCGAAG
gRNA4	TGTACCGGAGGTGATCGATC
gRNA5	CCGAAGATCCCAACGAGGAG
gRNA6	CTTTCCACCCAGTGACGACG

Table S2. Primers for E1A copy numbers determination.

Name	Sequence
Alb-primer	Forward: TTTGCAGATGTCAGTGAAAGAGA<br>Reverse: TGGGGAGGCTATAGAAAATAAGG
E1A-P1 primer	Forward: GAACCACCTACCCTTCACGA<br>Reverse: GCACCGCCAACATTACAGAG
E1A-P2 primer	Forward: CCGGTTTCTATGCCAAACCTTGT<br>Reverse: CGGGGTGCTCCACATAATCTAAC
E1A-P3 primer	Forward: GGTTTCTATGCCAAACCTTGTAC<br>Reverse: CTGCCCATAATTTTCACTTACTGT

Table S3. Change of E1A gene in JH293-C21-C14 cells.

Mutation type	Clones of T vector	Change of gRNA6 situation	percentage
1#1	T2/T8/T11/T14	3 bases deletion	4/12
1#2	T4/T7/T15/T17	6 bases deletion	4/12
1#3	T3/T16	9 bases deletion	2/12
1#4	T5/T13	No change	2/12

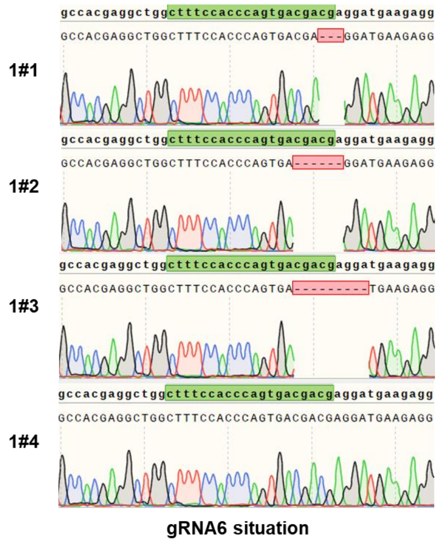

Figure S1. The deletion sequence of E1A gene in JH293-C21-C14 cells**.** The sequence highlighted in green represent the location of gRNA, 1#1 shows 3 nucleotides deletion, 1#2 shows 6 nucleotides deletion, 1#3 shows 9 nucleotides deletion, 1#4 shows no nucleotides deletion, 1#3 shows 9 nucleotides deletion. Bases with green background indicate the gRNA6 targeted sequence. Green single peak represented Adenine (A), blue single peak represented Cytosine (C), black single peak represented Guanine (G)and red single peak represented Thymine (T).

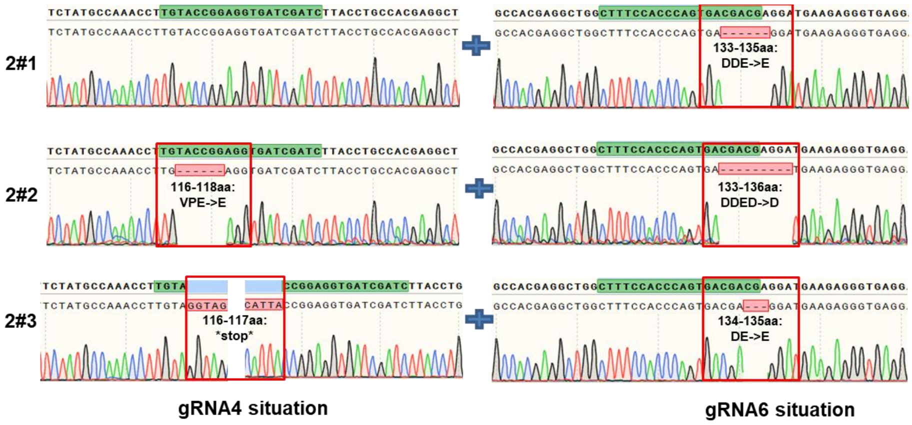

Figure S2. The deletion sequence of E1A gene in JH293-C21-C14-C28 cells**.** The sequence highlighted in green represent the location of gRNA6 or gRNA4, 2#1 shows 6 nucleotides deletion in gRNA6 situation only, 2#2 shows 9 nucleotides deletion in gRNA6 situation and 6 nucleotides deletion in gRNA4 situation, 2#3 shows 3 nucleotides deletion in gRNA6 situation and insertion mutation in gRNA4 situation.Bases with green background indicate the gRNA4 or gRNA6 targeted sequence. Green single peak represented Adenine (A), blue single peak represented Cytosine (C), black single peak represented Guanine (G)and red single peak represented Thymine (T).

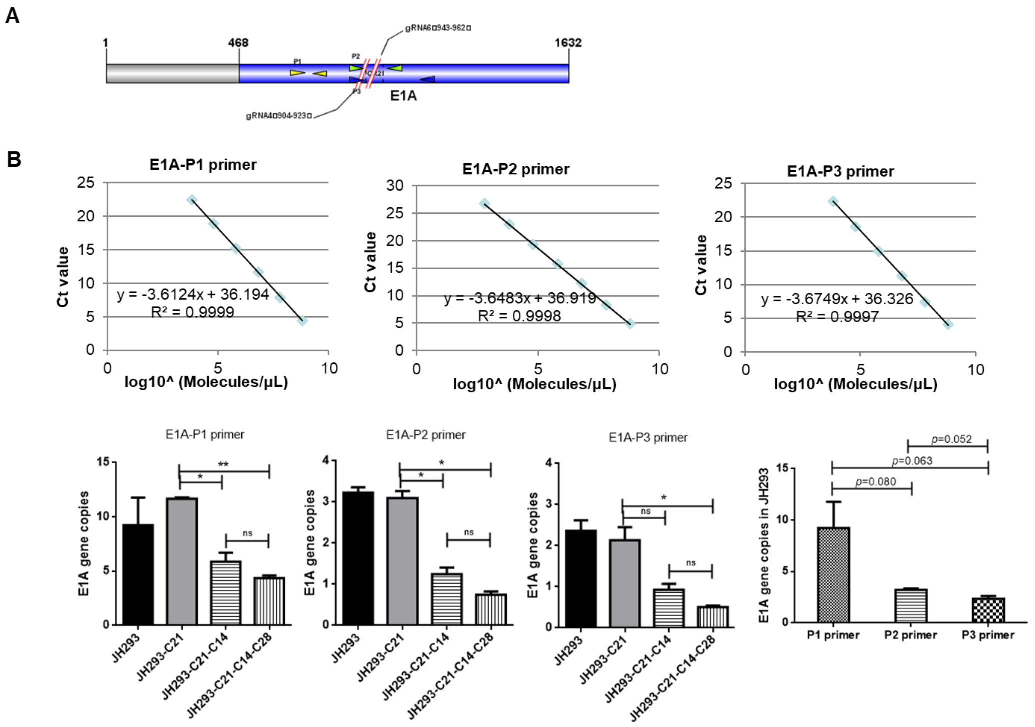

Figure S3. Detection of E1A copy numbers by using different primers. (A)The location of gRNA6, gRNA4, E1A-P1 primer, E1A-P2 primer and E1A-P3 primer in E1A gene. (B) The E1A copy numbers measured with E1A-P1 primer, E1A-P2 primer and E1A-P3 primer based on the respective standard curve. The bars represent the SE. The *p* values were obtained by one-tailed matched pair Student's *t* tests (* presents *p<0.05*, ** presents *p<0.005* and *ns* presents no significant difference compared with JH293-C21 group.
